# Differential expression profiles of lncRNAs and a preliminary study on the mechanism of lncRNA FAM225A in triple seronegative myasthenia gravis

**DOI:** 10.3389/fimmu.2025.1664131

**Published:** 2025-12-18

**Authors:** Yuehan Hao, Chen Chen, Bo Wang, ChunHua Yang, Ying Zhu, Ruixia Zhu

**Affiliations:** 1 Department of Neurology, The First Affiliated Hospital of China Medical University, Shenyang, China; 2 Key Laboratory of Neurological Disease Big Data of Liaoning Province, Shenyang, China; 3 Shenyang Clinical Medical Research Center for Difficult and Serious Diseases of the Nervous System, Shenyang, China; 4 The Research Center for Medical Genomics, Key Laboratory of Cell Biology, National Health Commission of the People’s Republic (PR) China, Key Laboratory of Medical Cell Biology, Ministry of Education of the People’s Republic (PR) China, School of Life Sciences, China Medical University, Shenyang, China

**Keywords:** immune infiltration, long non-coding RNA, miR-150-5p, T cell differentiation, triple-seronegative myasthenia gravis

## Abstract

**Background:**

Triple-seronegative (Triple-SN) myasthenia gravis (MG) is a subtype of MG, and its diagnosis and treatment are challenging. Our study aims to discover new biomarkers and potential therapeutic targets and explore the preliminary mechanisms of triple-SN MG.

**Methods:**

Peripheral blood mononuclear cells (PBMCs) were collected from 15 patients with triple-SN MG who were newly diagnosed with the disease and 15 healthy controls. Various experimental techniques and analysis methods, such as PBMC isolation, microarray analysis, dual-luciferase reporter assay, quantitative real-time polymerase chain reaction (qRT-PCR), cell culture, and transfection, were used.

**Results:**

Our study identified 385 differentially expressed genes (DEmRNAs) and 361 differentially expressed lncRNAs (DElncRNAs) in triple-SN MG. Notably, lncRNA FAM225A, one of the top five downregulated DElncRNAs, was verified to decreased and negatively correlated with the clinical severity of triple-SN MG. Functional enrichment analysis, immune infiltration analysis and further experiments revealed that FAM225A affected the imbalance of Th1/Th2 by targeting hsa-miR-150-5p in the pathogenesis of triple-SN MG.

**Conclusions:**

This study is the first to provide important clues for understanding the pathological mechanism of triple-SN MG, which might contribute to the discovery of novel diagnostic and therapeutic monitoring biomarkers and new targets for the treatment of triple-SN MG.

## Background

Myasthenia gravis (MG) is an immune disease regulated by antibodies that affect the postsynaptic membrane, further impairing neuromuscular transmission and leading to fatigable muscle weakness. MG is divided into four subgroups according to the different antibodies: acetylcholine receptor (AChR) MG, muscle-specific kinase (MuSK) MG, low-density lipoprotein receptor-related protein4 (LRP-4) MG, and triple-seronegative (triple-SN) MG (1, 2). Triple-SN MG accounts for 5%–7% of all MG cases, with a female predominance, and the most frequent initial symptoms are ptosis, diplopia, and generalized weakness (3). Triple-SN MG often presents a diagnostic and therapeutic challenge due to the limited understanding of the disease mechanism. Therefore, it is urgent to explore the detailed mechanism of triple-SN MG, providing novel biomarkers and potential treatment targets for triple-SN MG.

Long non-coding RNAs (lncRNAs) are endogenous molecules that regulate gene expression by dominating transcription, post-transcription, and chromatin modification. Emerging evidence has shown the dysregulation of lncRNAs in the pathogenesis of MG, particularly through their involvement in the activity of transcription factors, inflammatory response, leukocyte activation, and lymphocyte proliferation (4–6). LncRNAs show aberrant expression patterns that modulate T cell activation and proliferation. In addition, lncRNAs appear to participate in the regulation of the equilibrium of T helper (Th) 17 cells, regulatory T (Treg) cells, and Th1 cells (7–9) Additionally, lncRNAs may promote B cell proliferation and antibody production. IFNG-AS1, SNHG16, MALAT-1, GAS5, and XLOC_003810 have been found to be involved in the progression of MG (6, 10, 11). The expression of SNHG16 is increased in MG patients’ peripheral blood mononuclear cells (PBMCs) compared with that in controls, which act as a competing endogenous RNA (ceRNA) for let-7c-5p, thereby promoting Jurkat cell proliferation (12). Another dysregulated lncRNA, IFNG-AS1, is associated with MG severity and AChR antibody titer and regulates the balance of Th1/Treg cells (9). GAS5 is expressed at reduced levels in CD4+T cells, potentially disrupting the Treg/Th17 balance by targeting miR-23a (13). Above all, we speculate that lncRNAs may participate in the pathogenesis of triple-SN MG. Our research plan is to identify and validate key lncRNAs in triple-SN MG using comprehensive multi-omics analysis and experimental validation, and provide a novel perspective on the diagnosis, pathogenesis, treatment, and efficacy monitoring of triple-SN MG.

## Methods

### Patient collection

We collected data from 15 patients with first diagnosed triple-SN MG patients and 15 healthy controls matched for sex and age at the neurology department of the The First Affiliated Hospital of China Medical University. The inclusion criteria for patients were as follows (1): age between 18 and 80 years (2); first diagnosis of generalized MG, AChR, MuSK, or LRP-4 antibodies not detected in serum (3); had not yet received immunotherapy; and (4) no other immune system diseases. The exclusion criteria for patients included (1): patients with ophthalmic MG (2), had received corticosteroids or other non-steroidal immunosuppressants, and (3) with other immune system diseases. All participants provided written informed consent. This study was consented to the First Hospital of China Medical University ethics committee (2025–718–2). We randomly selected three cases from the patient and control groups for lncRNA microarray analysis. All samples from patients and controls were verified using quantitative real-time polymerase chain reaction (qRT-PCR).

### PBMC separation and microarray analysis

We isolated PBMCs from the peripheral blood using human lymphocyte separation medium (Solarbio, China) following the manufacturer’s instructions and extracted total RNA using TRIzol^®^ Reagent (Invitrogen, USA). NanoDrop^®^ ND-1000 was used to assess RNA quality and quantity. An Arraystar Human LncRNA Microarray (V5.0) was used to detect 39,317 lncRNAs and 21,174 protein-coding transcripts. Sample labeling and microarray hybridization were performed according to the experimental protocol of Agilent One-Color Microarray-Based Gene Expression Analysis (Agilent Technologies). The RNA integrity number (RIN) was used to measure RNA integrity, with a value greater than 7. For the fluorescent labeling array Cy3/Cy5, the doping rate of the labeled product was >1.5 pmol/μg. A built-in control probe in the array was used to monitor the hybridization efficiency. The background intensity of the non-probe area was set to a threshold below the 95% percentile of the global signal. Agilent Feature Extraction software (v11.0.1.1) was used to obtain microarray images and acquire raw data. GeneSpring GX v12.1 software (Agilent Technologies) was used for normalization and data handling. Quantile normalization was used to make the signal distribution consistent and eliminate technical bias. The Benjamini–Hochberg method was applied to correct the p-value, with a threshold set to FDR <0.05. After normalization and correction, we screened high-quality probes for further analysis.

### Methods of filtering differentially expressed lncRNAs and mRNAs

Differential analysis was performed between patients with triple-SN MG and controls using the limma package. The filtering standard of DElncRNAs and DEmRNAs was p <0.05 and fold change (FC) >1.5. Gradient volcano maps, radar charts, and circular heatmaps were used to visualize DEmRNAs and DElncRNAs using the ggplot2 package.

### Functional enrichment analysis

To confirm the biological significance of DEmRNAs, we performed Gene Ontology (GO) and Kyoto Encyclopedia of Genes and Genomes (KEGG) pathway analyses using the clusterProfiler package. Statistical significance was set at p <0.05, and results were visualized using the ggplot2 package.

### Gene set enrichment analysis

GSEA was conducted using the clusterProfiler and msigdbr packages to detect significantly enriched signaling pathways. The criteria were adjusted P <0.05, absolute NSE value >1, and FDR <0.05.

### Immune infiltration analysis

We used the CIBERSORT algorithm to infer the immune cell composition in patients with triple-SN MG. The enrichment score of immune cells in samples was calculated using ssGSEA algorithm to analyze the infiltration of immune cells in patients with triple-SN MG. Immune cell visualization and correlation analysis were performed using the ggplot2 package.

### Cell culture

The isolated PBMCs according to the above method were resuspended in RPMI-1640 medium (vivacell) supplemented with 10% FBS and 1% penicillin-streptomycin. Human embryonic kidney 293T cells (ATCC, Manassas, VA, USA) were cultured in high-glucose Dulbecco’s Modified Eagle Medium (DMEM) supplemented with 10% FBS, 1% penicillin-streptomycin, and 1 mM sodium pyruvate. Jurkat cells (Pricella Biotechnology Co., Ltd, Wuhan, China) were maintained in RPMI 1640 medium (vivacell) supplemented with 10% fetal bovine serum (FBS) and 1% penicillin/streptomycin. Cells were maintained at 37 °C in a humidified atmosphere of 5% CO_2_. The fresh medium was substituted for the old one every 2 or 3 days depending on the cell growth status.

### Cell transfection

Jurkat cells and PBMCs were cultured in six-well plates for 24 h prior to transfection. Upon reaching 60%–80% confluence, FAM225A-targeting siRNA (si2983-FAM225A) and overexpression (OE-FAM225A) were introduced into the cells using Lipofectamine 3000 (Invitrogen). Six hours post-transfection, the culture medium was replaced with a fresh medium. Subsequently, total RNA was isolated from the transfected cells for qRT-PCR analysis.

### Luciferase reporter assay

LncRNA FAM225A fragment containing the speculative binding site of hsa-miR-150-5p was constructed to psiCHECK2 (Tsingke) vector, namely FAM225A-WT. The sequence of the predicted binding site was mutated and constructed into the psiCHECK2 vector called FAM225A-MUT. FAM225A-WT or FAM225A-MUT vectors were co-transfected with hsa-miR-150-5p mimics or negative controls in 293T cells using Lipofectamine 3000 (Invitrogen). Luciferase activity was detected using the Dual-Luciferase^®^ Reporter Assay System (Promega).

### qRT-PCR

Total RNA was extracted from PBMCs or Jurkat cells using TRIzol reagent (Sigma), followed by cDNA synthesis using the HiScript III 1st Strand cDNA Synthesis Kit (Vazyme). Quantitative PCR was conducted using ChamQ Universal SYBR qPCR Master Mix (Vazyme) on a ViiA 7 Real-time PCR System (Applied Biosystems). The primer sequences are listed in **Supplementary Table S1**.

### Statistical analysis

We used SPSS 25.0 to conduct the statistical analysis. The variables of continuation are displayed as mean ± standard deviation (SD). Intergroup comparisons were evaluated using the Student’s t-test for two groups and one-way ANOVA for multiple groups. Pearson and Spearman correlation analyses were also conducted. A p-value of less than 0.05 was considered statistically significant.

## Results

### The clinical feature of triple-SN MG patients and controls

**Figure 1:**
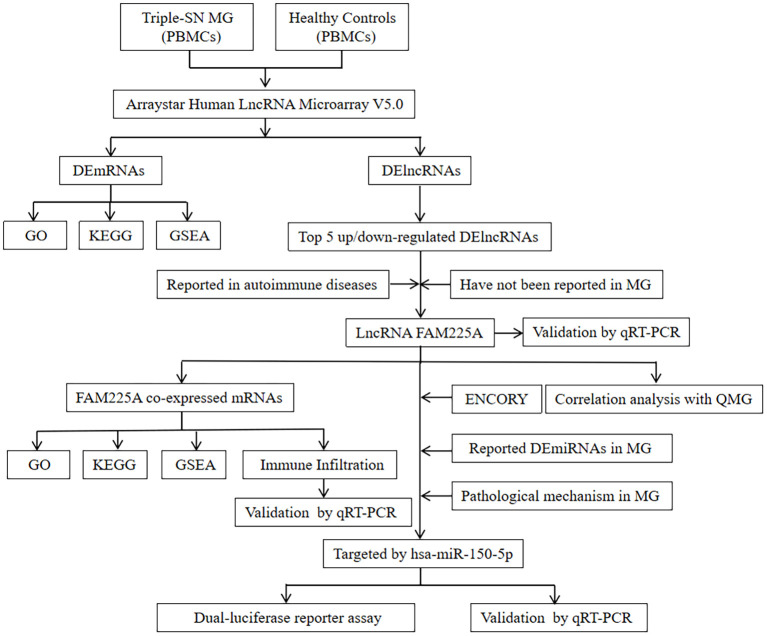
The flow-process diagram of this study. Triple-SN MG, triple-seronegative myasthenia gravis; PBMCs, peripheral blood mononuclear cells; DEmRNAs, differentially expressed genes; DElncRNAs, differentially expressed lncRNAs; GO, Gene Ontology; KEGG, Kyoto Encyclopedia of Genes and Genomes; GSEA, gene set enrichment analysis; MG, myasthenia gravis; qRT-PCR, real-time polymerase chain reaction; QMG, quantitative myasthenia gravis; DEmiRNAs, differentially expressed miRNAs.

Fifteen patients with triple-SN MG had an average age of 57.60 ± 15.06, and 15 healthy controls had an average age of 53.20 ± 12.85. There were no significant differences in age and sex (p <0.05). The clinical characteristics are shown in **Supplementary Table S2**. The flow process diagram is presented in **Figure 1**.

### Identification of DElncRNAs and DEmRNAs

**Figure 2:**
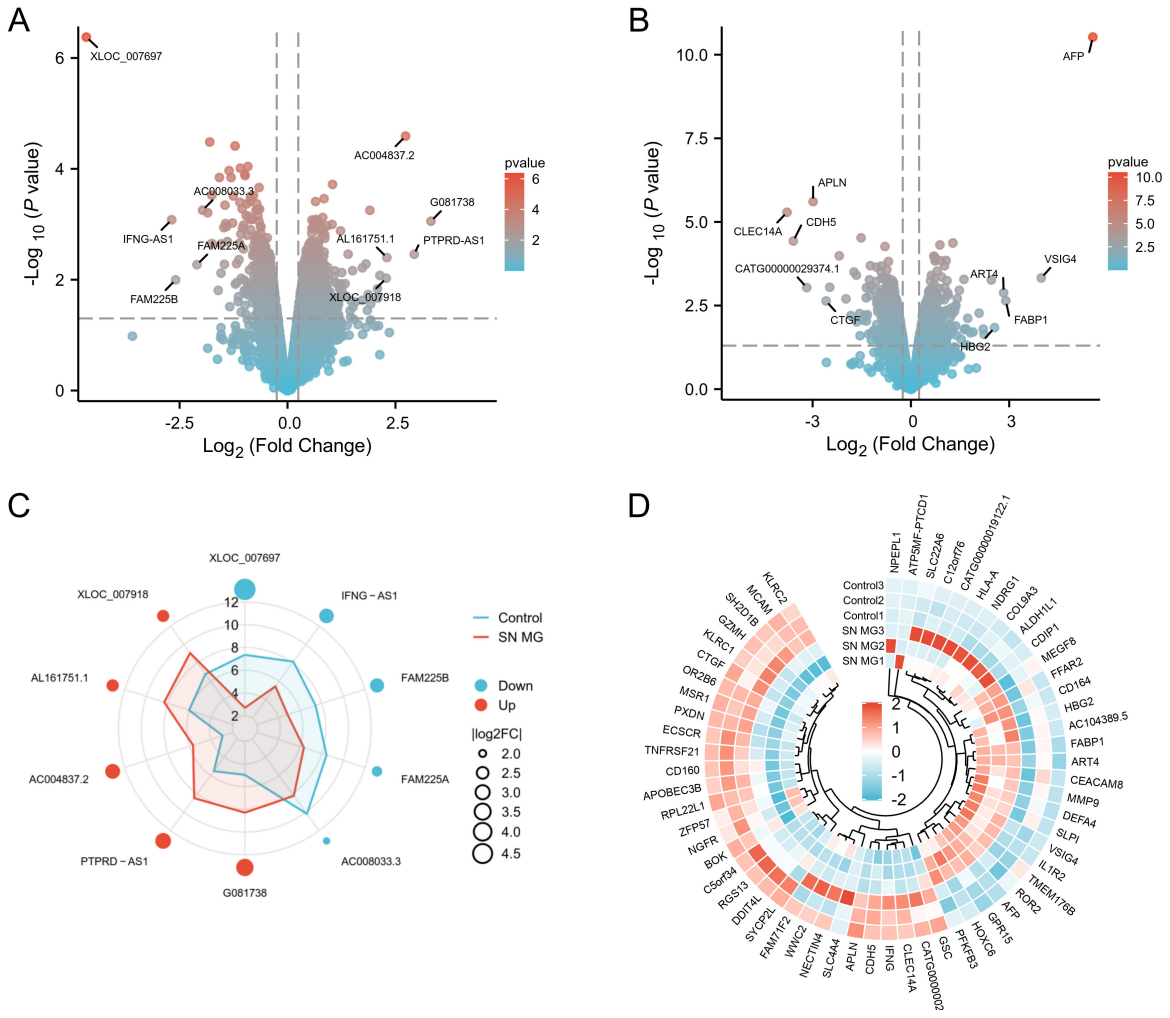
Identification of DElncRNAs and DEmRNAs in patients with triple-SN MG. **(A)** Gradient volcano map of DElncRNAs in triple-SN MG patients compared with controls. The top five upregulated and downregulated DElncRNAs were labeled. **(B)** Gradient volcano map of DEmRNAs in triple-SN MG patients compared with controls. The top five upregulated and downregulated DEmRNAs are labeled. **(C)** The top five upregulated and downregulated DElncRNAs are shown in a radar chart. **(D)** The top 30 upregulated and downregulated mRNAs are shown in a circular heatmap. Red and blue dots represent upregulation and downregulation, respectively.

After standardizing and processing the raw data using GeneSpring GX v12.1 software and removing low-quality probes, we obtained 14,622 DElncRNAs and 14,286 DEmRNAs. We then screened out 361 DElncRNAs (204 upregulated, 157 downregulated, **Supplementary Table S3**) and 385 DEmRNAs (201 upregulated, 184 downregulated, **Supplementary Table S4**) using the criterion of *p* < 0.05 and FC >1.5. The gradient volcano maps of DElncRNAs and DEmRNAs are shown in **Figures 2A, B**. To display DElncRNAs and DEmRNAs more intuitively, we used a radar chart to show the top five upregulated and downregulated DElncRNAs (**Figure 2C**) and a circular heatmap to show the top 30 upregulated and downregulated mRNAs (**Figure 2D**).

### Functional enrichment analysis of DEmRNAs in triple-SN MG

**Figure 3:**
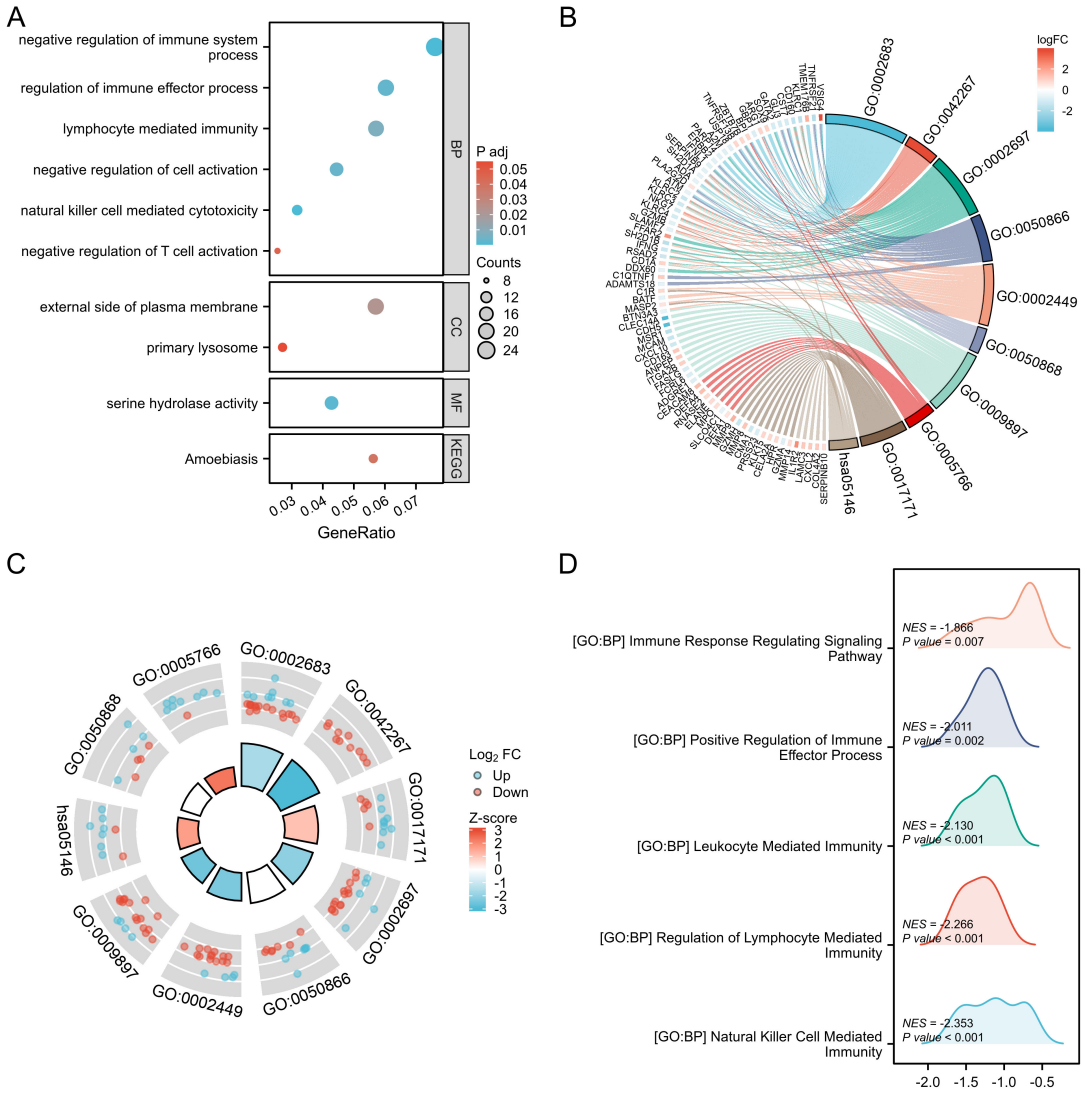
Functional enrichment analysis of DEmRNAs in triple-SN MG. **(A)** Representative functional enrichment analyses of GO and KEGG are shown in a bubble chart. **(B)** Representative functional enrichment analyses of GO and KEGG are shown in a chordal graph with log2FC. **(C)** Representative functional enrichment analyses of GO and KEGG are shown in a cyclic graph with Z-scores. **(D)** Representative signal pathways identified by GSEA are shown in a mountain range map.

The representative functional enrichment analyses of GO and KEGG are shown in **Figures 3A–C**, including natural killer cell-mediated cytotoxicity, negative regulation of cell activation, lymphocyte-mediated immunity, regulation of immune effector processes, negative regulation of immune system processes, and negative regulation of T cell activation. To further clarify the signaling pathways related to triple-SN MG, we conducted GSEA and found that natural killer cell-mediated immunity, leukocyte-mediated immunity, regulation of lymphocyte-mediated immunity, immune response regulating signaling pathway, and positive regulation of immune effector process played important roles (**Figure 3D**).

### LncRNA FAM225A was downregulated in triple-SN MG and was associated with clinical scores

**Figure 4:**
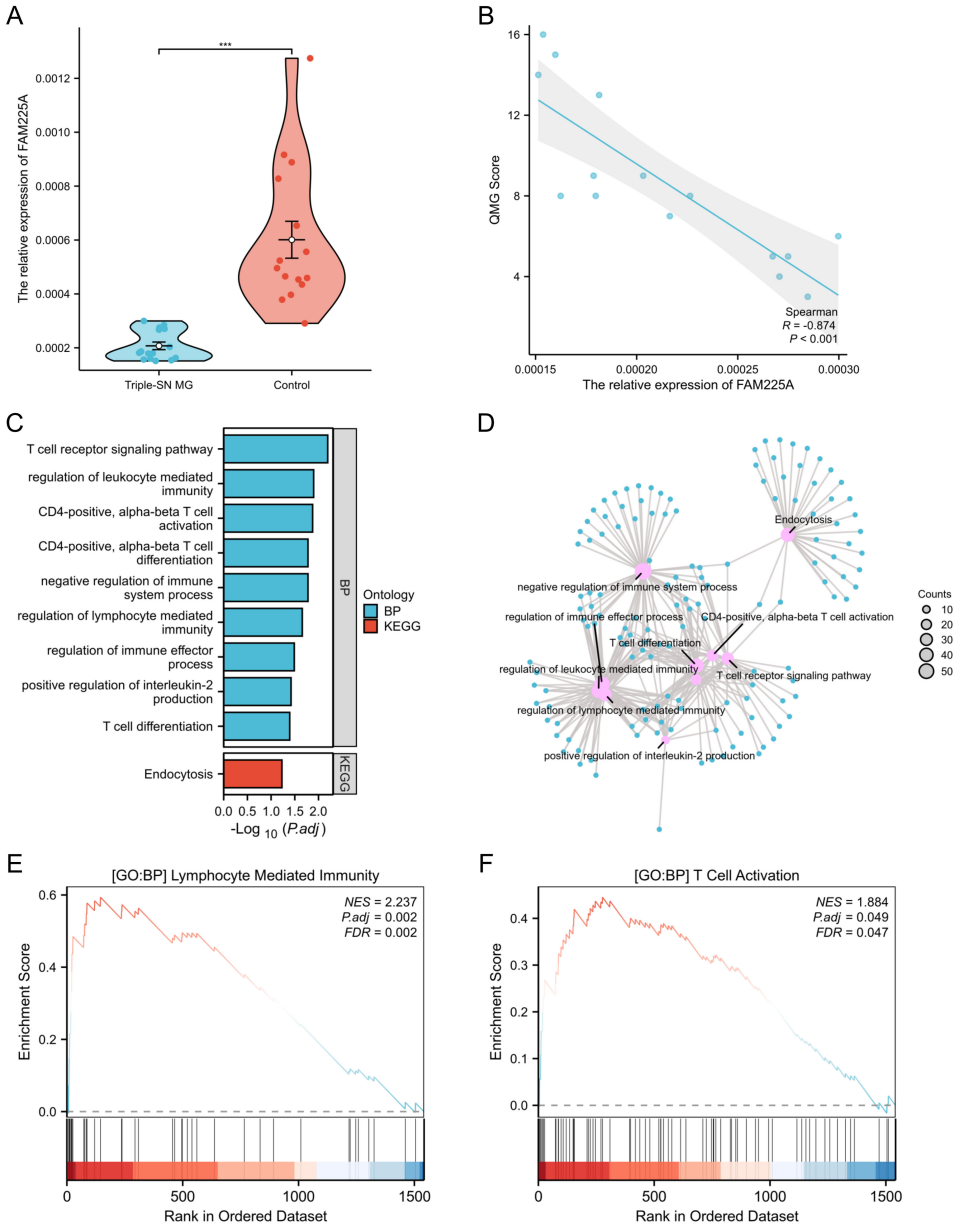
Verification of the relative expression of FAM225A in patients with triple-SN MG and the clinical significance and functional analysis of FAM225A. **(A)** Relative expression of FAM225A in PBMCs of patients with triple-SN MG and controls using qRT-PCR. ***p <0.001. **(B)** Scatter plot of the correlation between the relative expression of FAM225A in patients with triple-SN MG and QMG score. **(C)** Bar chart showing representative functional enrichment analyses of GO and KEGG for FAM225A co-expressed mRNAs. **(D)** Network diagram showing representative functional enrichment analyses of GO and KEGG for FAM225A co-expressed mRNAs. **(E)** The results of GSEA displayed lymphocyte-mediated immunity was significantly enriched. **(F)** The results of GSEA displayed T cell activation was significantly enriched.

To explore novel lncRNA biomarkers in patients with triple-SN MG, we searched for the top five upregulated and downregulated lncRNAs (**Figure 2C**) in PubMed. We found that IFNG-AS1 and FAM225A were reported in autoimmune diseases, XLOC_007697, FAM225B, and PTPRD-AS1 were in tumors, and AC008033.3, G081738, AC004837.2, AL161751.1, and XLOC_007918 were in no articles (14–29). Nevertheless, the mechanism of IFNG-AS1 in MG has already been studied; therefore, we further explored the role of lncRNA FAM225A in triple-SN MG (9, 30). FAM225A was downregulated in triple-SN MG compared with controls according to the results of our microarray. We verified FAM225A expression in the PBMCs of 15 patients with triple-SN MG and 15 controls using qRT-PCR. Our study showed that FAM225A was significantly downregulated (*p <*0.001, **Figure 4A**), which was consistent with our microarray data. To further clarify the association of FAM225A and clinical scores, we conducted correlation analysis between quantitative myasthenia gravis (QMG) score and the relative expression of FAM225A, and found that FAM225A in triple-SN MG patients was negative correlation with QMG score (*R* = −0.874, p-value <0.001, **Figure 4B**). In addition, to reveal the function of FAM225A in triple-SN MG, we conducted functional enrichment analysis of FAM225A co-expressed mRNAs using the standard of *p <*0.05 and Pearson’s correlation coefficients |r| >0.8 (**Supplementary Table S5**). The representative functional enrichment analyses of GO and KEGG are shown in **Figures 4C, D**, including the regulation of lymphocyte-mediated immunity, positive regulation of interleukin-2 production, CD4 positive T cell activation and differentiation, T cell receptor signaling pathway, and negative regulation of immune system process. The results of GSEA showed that lymphocyte-mediated immunity and T cell activation were significantly enriched (**Figures 4E, F**).

### Characteristics of immune cells distribution in triple-SN MG patients

**Figure 5:**
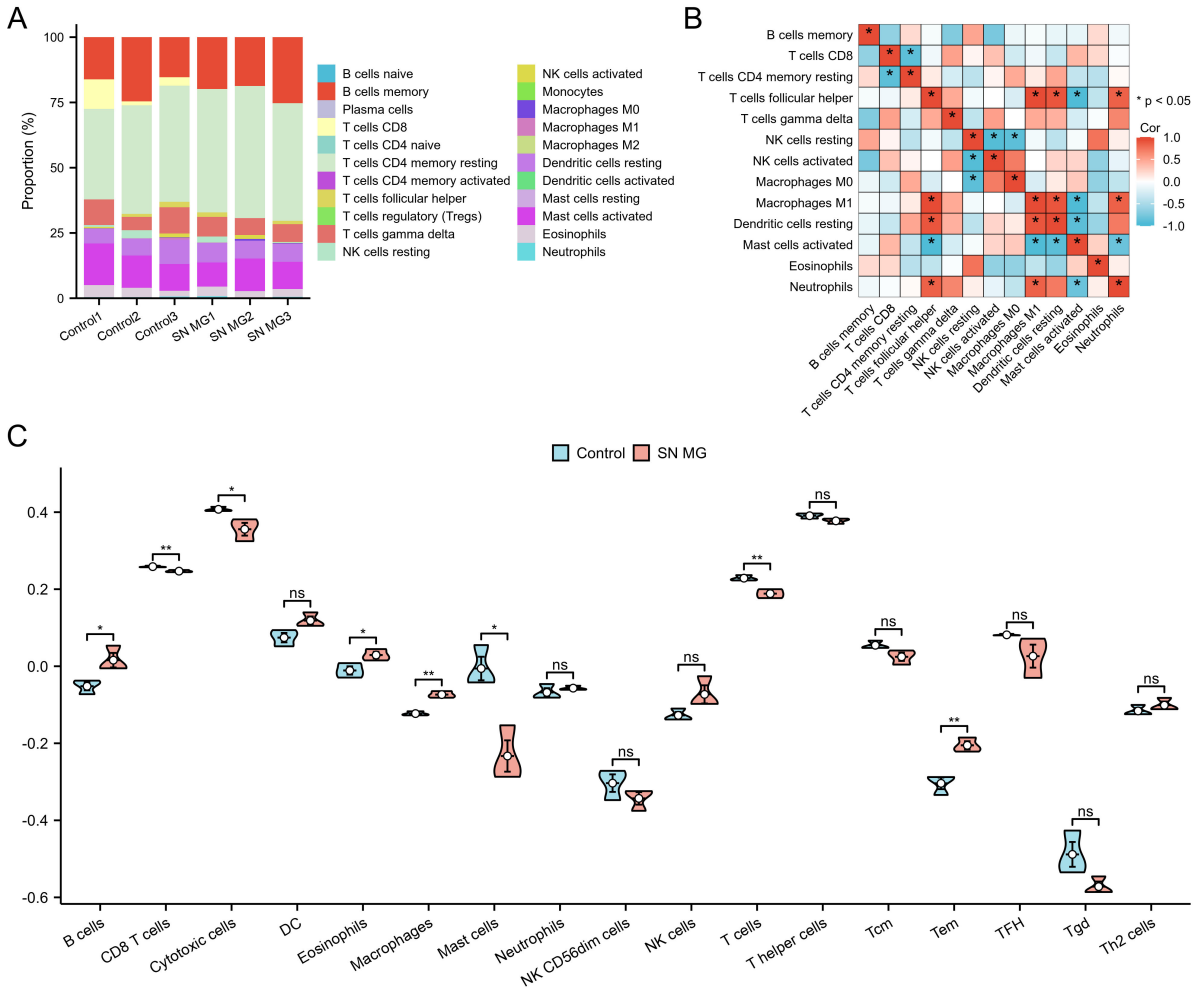
Immune cell infiltration analysis of FAM225A co-expressed mRNAs in patients with triple-SN MG and controls. **(A)** Relative proportions of different immune cells in patients with triple-SN MG and controls using the CIBERSORT algorithm. **(B)** The correlation heatmap displays the interrelationships among different immune cell types. **(C)** Violin plot displaying the difference in enrichment score of infiltrating immune cells between patients with triple-SN MG and controls using the ssGSEA algorithm. *p <0.05, **p <0.01.

To explore the mechanism of FAM225A in the immune process of triple-SN MG, we conducted an immune cell infiltration analysis of FAM225A co-expressed with mRNAs. The proportions of different immune cells in patients with triple-SN MG and controls, as determined using the CIBERSORT algorithm, are shown in **Figure 5A**. The correlation analysis of different immune cells displayed a negative correlation of T cells CD8 and CD4 memory resting, and a positive correlation between Macrophages M1 and T cells (p <0.05, **Figure 5B**). Additionally, immune infiltration analysis using the ssGSEA algorithm showed that CD8 T cells, cytotoxic cells, mast cells, and T cells were significantly decreased, while B cells, eosinophils, macrophages, and Tem were increased significantly in triple-SN MG compared with controls (**Figure 5C**).

### LncRNA FAM225A was a target of hsa-miR-150-5p

**Figure 6:**
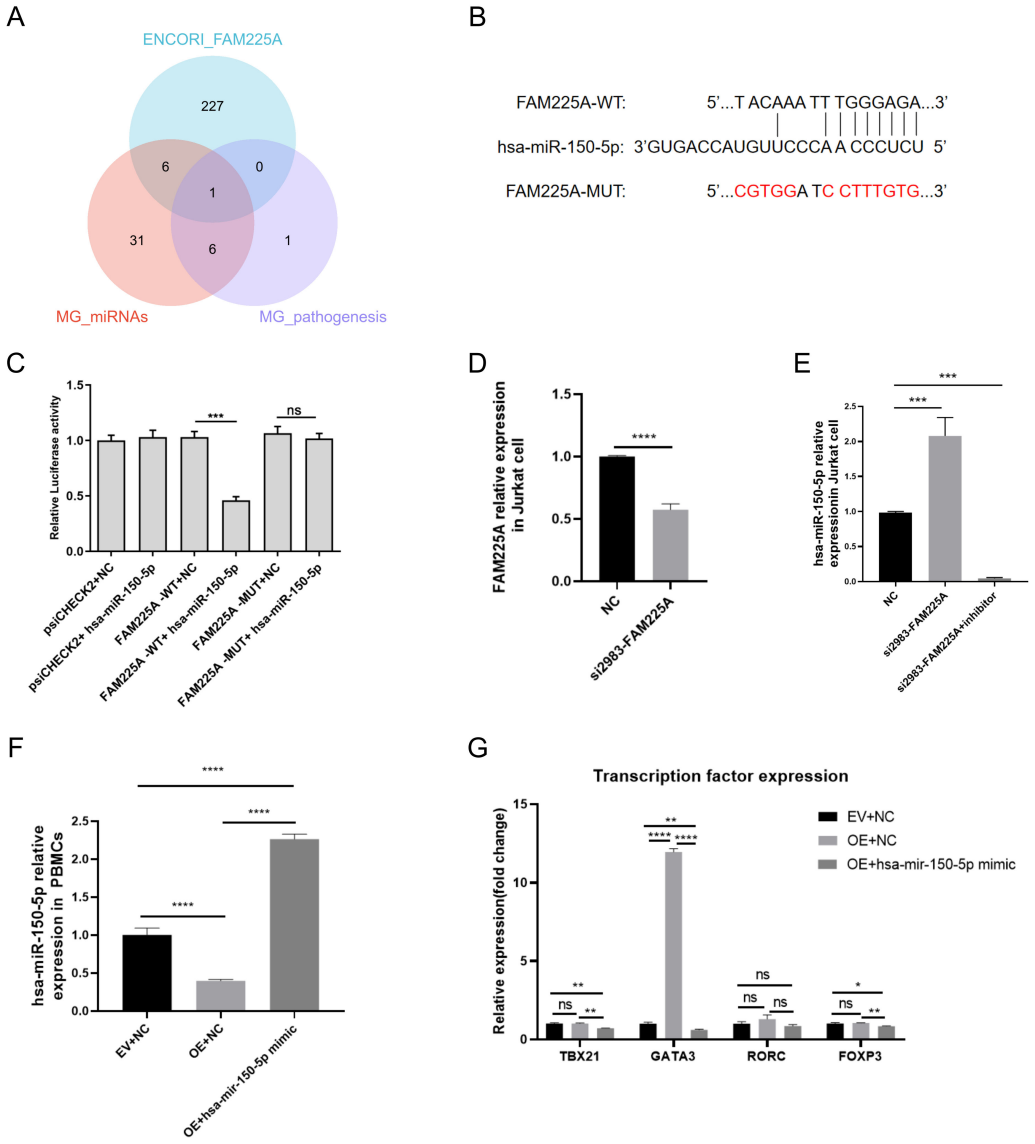
LncRNA FAM225A is a target of hsa-miR-150-5p. **(A)** The Venn diagram shows the intersection of predicted miRNAs in the ENCORY database, reported differentially expressed miRNAs in patients with MG, and miRNAs that have been proven to participate in the pathological mechanism of MG. **(B)** Putative hsa-miR-150-5p binding sequences of FAM225A-wild type (WT) and FAM225A-mutated type (MUT). **(C)** The luciferase reporter plasmid containing FAM225A-WT or FAM225A-MUT was co-transfected with the hsa-miR-150-5p mimic or miRNA NC into 293 T cells. Luciferase activity was calculated as the ratio of firefly to Renilla activity. **(D)** Relative expression of FAM225A in Jurkat cells after transfection with NC and si2983-FAM225A, as measured by qRT-PCR. **(E)** Relative expression of hsa-miR-150-5p in Jurkat cells after transfection with NC, si2983-FAM225A, and si2983-FAM225A in combination with miR-150-5p inhibitor, as measured by qRT-PCR. **(F)** Relative expression of hsa-miR-150-5p in PBMCs after transfection with NC, OE-FAM225A, and OE-FAM225A in combination with miR-150-5p mimic, as measured by qRT-PCR. **(G)** Relative expression of transcription factors related to Th1 (TBX21), Th2 (GATA3), Th17 (RORC), and Treg cells (FOXP3) by qRT-PCR. **p < 0.01, ***p < 0.001, ****p < 0.0001.

To obtain miRNAs that may target lncRNA FAM225A, we took the intersection of predicted miRNAs in the ENCORY database, reported differentially expressed miRNAs in MG (5), and miRNAs that had been proven to participate in the pathological mechanism of MG (**Figure 6A**) (6). Hsa-miR-150-5p was the intersecting miRNA, which was speculated to target FAM225A (**Supplementary Table S6**). Bioinformatics analysis identified a putative hsa-miR-150-5p binding motif within the FAM225A sequence. To experimentally validate this interaction, we engineered luciferase reporter vectors (psiCHECK2) containing either wild-type (WT) or mutated (MUT) FAM225A sequences (**Figure 6B**). The results displayed that the luciferase activity of FAM225A-WT + hsa-miR-150-5p group was significantly decreased compared to that of the FAM225A-WT + NC group in 293 T cells, indicating that hsa-miR-150-5p might target FAM225A (*p* < 0.001, **Figure 6C**). In addition, no significant variation was found in luciferase activity of the FAM225A-MUT + hsa-miR-150-5p group compared to the FAM225A-MUT + NC group, suggesting that after the binding sequences were mutated, hsa-miR-150-5p no longer targeted FAM225A (**Figure 6C**). The identified “UACAAAUUUGGGAGA” sequence represents a functional hsa-miR-150-5p binding.

We further verified the regulatory relationship between FAM225A and hsa-miR-150-5p in Jurkat cells and in PBMCs. qRT-PCR was used to check transfection efficiency when FAM225A was knocked down by si2983-FAM225A transfection in Jurkat cells. The results showed that the relative expression of FAM225A was significantly decreased when si2983-FAM225A was transfected into Jurkat cells (p <0.0001, **Figure 6D**). In addition, the relative expression of hsa-miR-150-5p was significantly increased when si2983-FAM225A was transfected into Jurkat cells, whereas the miR-150-5p inhibitor blocked this increase (p <0.001, **Figure 6E**). The significant decrease in miR-150-5p in the inhibitor group might be due to strong antisense binding, a known limitation of qPCR quantification of miRNAs under inhibitor treatment. In addition, we verified the regulatory relationship between FAM225A and hsa-miR-150-5p in PBMCs. OE-FAM225A (the overexpression of FAM225A), OE-FAM225A in combination of miR-150-5p mimic, and NC was transfected in PBMCs, respectively. The results showed that the relative expression of hsa-miR-150-5p was decreased in the OE-FAM225A group, whereas miR-150-5p mimic significantly reversed this decline (p <0.0001, **Figure 6F**). These results verified the regulatory relationship between FAM225A and hsa-miR-150-5p.

### FAM225A alters the differentiation of T cell subsets *in vitro*

In the immune microenvironment, different CD4+T cell subsets exert pro-inflammatory or anti-inflammatory effects. The results of the immune infiltration analysis suggested that FAM225A may affect T cell differentiation. To further validate the above results, we compared the expression of related transcription factors of Th1, Th2, Th17, and Treg cells in the OE-FAM225A group and control, and observed whether the miR-150-5p mimic could reverse the above results using qRT-PCR. We found that the expression of Th2-related transcription factors (GATA3) was significantly increased and the Th1/Th2 ratio decreased in the OE-FAM225A group compared with the control, while there were no significant changes in the levels of transcription factors related to Th1 (TBX21), Th17 (RORC), and Treg cells (FOXP3) (p <0.0001, **Figure 6G**). In addition, the OE-FAM225A + miR-150-5p mimic group significantly blocked the increase in Th2-related transcription factors (p <0.0001, **Figure 6G**). The results indicate that FAM225A mainly affects Th2 cell differentiation and the imbalance of Th1/Th2 by targeting miR-150-5p in triple-SN MG.

## Discussion

MG is an acquired immunity disorder that causes abnormal transmission of the neuromuscular junction, leading to muscle weakness, significantly affecting patients’ daily life and work capability, and producing a burden on both individuals and society. Triple-SN MG is a special type of MG that often cannot be diagnosed and treated promptly due to the lack of explicit diagnostic biomarkers and limitations in treatment, with some patients experiencing poor treatment outcomes and frequent relapses. Thus, this study is of great significance in deepening the understanding of clinical biomarkers and pathogenesis, and exploring new diagnostic and therapeutic approaches for triple-SN MG.

Emerging evidence has implicated lncRNAs in the pathogenesis of diverse immune-mediated disorders (14–22). Currently, studies have summarized the dysregulation lncRNAs in the pathogenesis of MG, including IFNG-AS1, SNHG16, MALAT-1, GAS5, and XLOC_003810 (6, 10, 11). Among them, IFNG-AS1 is correlated with the level of anti-AchR antibodies and the severity of MG (9). However, the association between lncRNAs and triple-SN MG is not yet been yet fully understood. This study aimed to explore the expression changes and potential roles of lncRNAs in triple-SN MG, demonstrating the innovation and potential value of this research. We obtained 361 DElncRNAs in triple-SN MG through microarray analysis with the criteria of p <0.05 and FC >1.5, of which 204 DElncRNAs were significantly upregulated and 157 were significantly downregulated. To explore novel lncRNA biomarkers in patients with triple-SN MG, we searched the top five upregulated and downregulated lncRNAs in PubMed and found that lncRNA FAM225A was associated with autoimmune diseases and was not differentially expressed in antibody-positive patients with MG (9, 14–31). LncRNA FAM225A participated in systemic lupus erythematosus and some tumors via sponging miRNAs (23, 25, 32–36). However, its role in triple-SN MG-type has not yet been elucidated.

In this study, we found and validated that FAM225A was significantly decreased in triple-SN MG, suggesting that it may act as a potential regulator of triple-SN MG progression. To explore the potential functions of FAM225A in triple-SN MG, we conducted a functional enrichment analysis of co-expressed mRNAs of FAM225A. The results showed that the regulation of lymphocyte-mediated immunity was significantly enriched in the high-risk group. To further investigate the immune mechanism of FAM225A, we conducted an immune infiltration analysis using two algorithms. Bioinformatics analysis results indicated that FAM225A might play crucial roles in the immune mechanism of triple-SN MG by regulating T cell differentiation. T cells, including follicular helper T cells (Tfh), T helper 17 cells (Th17), and Tregs, participate in the immune mechanism of MG (37). However, there are few reports on the pathophysiology of T cells in triple-SN MG. Yuumi et al. elucidated that Tregs were downregulated and associated with disease severity in refractory SN MG patients (38). Tregs could inhibit effector T cells which regulate immune response, mainly by restraining antigen-presenting cells or by generating inhibitory cytokines (39). The exact immune mechanisms of different T cell subtypes in triple-SN MG remain unclear. In addition, MG is thought to be an autoimmune disease dependent on CD4+ T cells (40–42). Notably, CD8+ T cells were significantly downregulated by immune infiltration analysis in our study. Previous research revealed that CD8+ T cells were associated with thymic engraftment and secretion of pro-inflammatory cytokine IL-17 and IFNγ (41–43). Insufficient attention has been paid to this cell subtype in triple-SN MG. Our bioinformatics analysis results provide a broad research perspective on the mechanisms of different T cell subsets in triple-SN MG. Moreover, our study indicated that effector memory T (Tem) cells were aberrantly upregulated in triple-SN MG, consistent with the study by Huang et al. on MG, in which CD4+ Tem cells increased significantly (44). Tem cells can recognize pathogenic antigens, participate in initiating immune responses, and are involved in pathological immune processes by exerting inflammatory cytokines (45–50). A previous study pointed out that CD4+ Tem cells generate IL-17 when encountering antigens and regulate the balance of Th17/Th1 (51). However, the exact immune mechanism of Tem cells in triple-SN MG has not been clearly interpreted. Due to the extremely low incidence of triple-SN MG, clinically available blood samples are extremely limited, and it is temporarily impossible to extract sufficient fresh cells for flow cytometry. Meanwhile, unlike experimental autoimmune myasthenia gravis (EAMG) in AChR-positive MG (52), there is currently a lack of cell and animal models for triple-SN MG due to the lack of specific antibodies. To further validate the results of functional enrichment and immune infiltration analysis, we compared the expression of related transcription factors of Th1 (TBX21), Th2 (GATA3), Th17 (RORC), and Treg (FOXP3) cells in the OE-FAM225A group and control. We found that the expression of Th2-related transcription factors (GATA3) was significantly increased and the Th1/Th2 ratio decreased in the OE-FAM225A group, indicating FAM225A mainly affected Th2 cell differentiation and the imbalance of Th1/Th2, which might provide new insights into the study of the immune mechanism of triple-SN MG; however, further systematic experimental verification is required. In future research, we plan to gradually validate our findings through multicenter collaborations or the new development of *in vitro* models.

In our study, we predicted that miR-150-5p may target FAM225A through bioinformatics analysis and validated the interaction using a dual-luciferase reporter assay and qRT-PCR. miR-150-5p is a regulator that mediates the proliferation, activation, and differentiation of immunocytes and participates in the immune inflammatory response (53–57). Several studies have reported that miR-150-5p is closely associated with the regulation of T cell immune homeostasis in autoimmune diseases (53, 58). Cron et al. found that miR-150-5p mediates the survival state of CD8+ and CD4+ T cells via apoptosis-related pathways (58), and that miR-150-5p influences the balance of Th1/Th2 by promoting differentiation from Th0 cells to Th2 cells (59). Other studies have revealed that miR-150-5p increases IL-10 and decreases IL-17 levels, thereby affecting the immune process in MG (60). In summary, abnormal expression of miR-150-5p could cause autoimmune disorders by affecting T lymphocytes (61, 62). Thus, we speculated that FAM225A might regulate T cell differentiation by targeting miR-150-5p. In this study, we found that the OE-FAM225A + miR-150-5p mimic group significantly blocked the increased expression of the Th2-related transcription factor (GATA3) in the OE-FAM225A group, illustrating that FAM225A mainly affected Th2 cell differentiation by targeting miR-150-5p and exerting anti-inflammatory effects in Triple-SN MG. Further exploration is needed to comprehensively reveal the molecular mechanisms underlying triple-SN MG. In addition, ceRNA may be one of the potential modes of action of FAM225A in triple-SN MG, but not the only mechanism. We only explained its partial function; other mechanisms by which FAM225A may participate in triple-SN MG require further investigation to clarify its key role in disease occurrence and development. Our study provides a foundation for subsequent research.

One innovation of this study was the analysis of the relationship between FAM225A and the clinical characteristics of patients with triple-SN MG, through an analysis of the correlation between FAM225A and QMG scores to evaluate severity. We found that FAM225A was negatively correlated with the severity of clinical symptoms, suggesting that FAM225A may become a new biomarker for diagnosis and curative effect inspection, and a new target for treatment of triple-SN MG. Applying these results to clinical diagnosis and treatment requires further clinical research and validation, as well as comprehensive analysis based on the actual clinical situation. Further research on its mechanism and clinical value will contribute to the development of more effective treatment strategies. The clinical significance of the interaction between FAM225A and hsa-miR-150-5p in triple-SN MG requires further exploration to clarify its potential value in diagnosis, treatment, and prognosis.

This study had some limitations. First, the relatively small sample size might have affected generalizability of the results and made it difficult to fully reflect the overall situation of patients with triple SN-MG. Second, we did not conduct further experiments because of the extremely low incidence of triple-SN MG, the limited availability of clinically available blood samples, and the lack of cell and animal models for triple-SN MG. Although bioinformatics analysis and preliminary *in vitro* experimental results provide clues for understanding the pathogenesis of triple-SN MG, further *in vivo* experiments and clinical validation are still needed to ensure its reliability and effectiveness in clinical practice.

## Conclusions

This study is the first to conduct a microarray analysis of PBMCs from patients with triple-SN MG, revealing a series of immune-related molecular mechanisms in triple-SN MG. LncRNA FAM225A was expected to become a clinical biomarker, efficacy monitoring indicator, and potential therapeutic target for triple-SN MG. Our bioinformatics analysis results and preliminary *in vitro* validation suggested that FAM225A mainly affects Th2 cell differentiation and the imbalance of Th1/Th2 by regulating miR-150-5p. These results provide key clues for further exploration of the pathogenesis of triple-SN MG and open new avenues for future diagnosis and treatment. However, as mentioned above, this study has some limitations, and further sample size expansion, *in vivo* experiments, and clinical validation are needed in subsequent studies to promote clinical application.

## Data Availability

The raw data supporting the conclusions of this article will be made available by the authors, without undue reservation.
